# Expression of brain derived-neurotrophic factor and granulocyte-colony stimulating factor in the urothelium: relation with voiding function

**DOI:** 10.1186/s12894-015-0036-3

**Published:** 2015-05-08

**Authors:** Seung Mo Yuk, Ju Hyun Shin, Ki Hak Song, Yong Gil Na, Jae Sung Lim, Chong Koo Sul

**Affiliations:** Department of Urology, Korea St. Mary’s Hospital, College of Medicine, The Catholic University of Korea, Seoul, South Korea; Department of Urology, Korea Chungnam National University Hospital, College of Medicine, Chungnam National University, Daejeon, South Korea

**Keywords:** Bladder, Brain derived-neurotrophic factor, Granulocyte-colony stimulating factor, Overactivity

## Abstract

**Background:**

We designed this experiment to elucidate the relationship between the expression of brain derived-neurotrophic factor (BDNF), the expression of granulocyte-colony stimulating factor (G-CSF), and the development of overactive bladder (OAB). In our previous study, the urothelium was observed to be more than a simple mechanosensory receptor and was found to be a potential therapeutic target for OAB. Moreover, neuregulin-1 and BDNF were found to be potential new biomarkers of OAB. Here, we investigated the relationship between changes in the voiding pattern and the expression of BDNF and G-CSF in the urothelium and evaluated the effects of 5-hydroxymethyl tolterodine (5-HMT) on rats with bladder outlet obstruction (BOO).

**Methods:**

A total of 100 Sprague–Dawley rats were divided into the following groups: 20 control rats; 40 BOO rats; and 40 BOO rats administered 5-HMT (0.1 mg/kg). After BOO was induced for 4 weeks, the rats were assessed by cystometrography. The changes in BDNF and G-CSF expression were examined in both separated urothelial tissues and in cultured urothelial cells by reverse transcription polymerase chain reaction (RT-PCR).

**Results:**

BOO rats showed increased non-voiding activity [NVA; (number/10 voidings)] and bladder weight and decreased micturition volume (MV), micturition interval (MI), and micturition time (MT) relative to the controls. Moreover, the 5-HMT administration rats showed decreased NVA and bladder weight and increased MV and MI in comparison to the BOO rats. BDNF and G-CSF expression was increased in BOO rats and decreased following 5-HMT administration. In this model, voiding dysfunction developed as a result of BOO. As a therapeutic agent for OAB, the administration of 5-HMT improved the voiding dysfunction.

**Conclusions:**

BDNF and G-CSF might modulate voiding patterns through micturition pathways and might be involved only in the urothelium. Moreover, the expression of both genes in the urothelium might be related to voiding dysfunction in OAB patients. Thus, the urothelium has an important role in the manifestation of voiding symptoms.

## Background

Overactive bladder (OAB) is a clinical syndrome that is characterized by the presence of urinary urgency, with or without urgency incontinence, and is usually accompanied by daytime frequency and nocturia in the absence of proven infection or other obvious pathologies [1]. Bladder outlet obstruction (BOO) results in changes in bladder structure and function that include detrusor hypertrophy, elevated detrusor contractile pressure, and detrusor instability, which can result in OAB [2]. Prolonged BOO can also influence the occurrence and severity of OAB by increasing the production of nerve growth factors (NGF) and thereby inducing neuronal enlargement [3].

The urothelium has specialized sensory and signaling properties through which it responds to the chemical and physical environment and engages in reciprocal chemical communication with neighboring nerves in the bladder wall. The detrusor muscle has traditionally been known for having a significant influence on voiding function. However, the urothelium also plays an important role in this process, as evidenced by the effect of changes in urothelial characteristics on the regulation of voiding function. To elucidate the role of the urothelium in voiding function, we have focused solely on this tissue in the absence of the detrusor muscle. In our previous report, we suggested that urothelium-expressed neuregulin-1 and BDNF are potential new biomarkers of OAB [4]. Based on these findings, we believe that the urothelium is more than a simple mechanosensory receptor and may be a potential therapeutic target for OAB treatment. The present study builds upon the work from our previous study by evaluating the relation between voiding dysfunction and the expression of growth factors.

Previous studies has discovered a significant link between BDNF and OAB [4, 5]; however, little is known about whether a significant relationship exists between G-CSF and OAB. Fesoterodine (Pfizer Central Research, Sandwich, UK) is an antimuscarinic drug approved for the treatment of OAB, and is hydrolyzed by a nonspecific esterase to 5-hydroxymethyl tolterodine (5-HMT) [6]. 5-HMT is the active metabolite and is responsible for the antimuscarinic activity of this drug [6]. Antimuscarinics have been found to act in response to muscarinic receptors on detrusor smooth muscle cells to reduce spontaneous myocyte activity during the storage phase [7], thus eventually decreasing the frequency and intensity of detrusor contractions. Hence, the use of 5-HMT as a therapeutic agent of OAB can be used to confirm the presence of OAB, examine the role of BDNF and G-CSF in OAB, and evaluate changes in the urothelium. Here, we evaluated BDNF and G-CSF expression in a BOO rat model and the effect of 5-HMT on their expression both in the urothelium and in cultured urothelial cells (UCs) to understand the relationship between the expression of BDNF and G-CSF and voiding dysfunction. We aimed to test whether 1) OAB occurrence and treatment are related to the BDNF and G-CSF expression, 2) 5-HMT would affect the expression of growth factors on the urothelium, and 3) the urothelium functions as more than a simple mechanosensory receptor in regulating bladder functions.

## Methods

### Animals

Female Sprague–Dawley rats (250–300 g; Daehan Biolink Co. Ltd, Daejeon, Korea) were used in this study. The experimental protocol was approved by the Animal Ethics Committee of the University of Chungnam, South Korea. The rats were handled according to the NIH guidelines. The sample size needed for evaluating the expression of the different growth factors, as affected by BOO, was determined. To achieve a power of 0.8, alpha value of 0.05, and sample size rate of 2 for detecting statistical differences, 11 animals would be required as controls.

Group I (20 rats) was the control group, group II (40) comprised BOO rats, and group III (40) comprised BOO rats injected with 5-HMT (0.1 mg/kg) in the rat tail vein (2 times/weeks) for 3 weeks. These doses of 5-HMT generally correspond to the doses used clinically in humans [8]; the 5-HMT was generously provided by Pfizer. The BOO rats were injected with 5-HMT as described by Melman et al. [9]. For inducing BOO, a lower midline incision was made to approach the bladder of each anesthetized rat and to expose the proximal urethra. A 3–0 polypropylene suture was used to tie the proximal urethra with a 24-G angioneedle sheath. After suturing, the angioneedle sheath was removed, leaving the urethra partially obstructed.

### Cystometry investigations

The BOO rats were anesthetized 4 weeks after they were injected with 5-HMT. Polyethylene catheters were implanted through the bladder dome and exteriorized at the scapular level. A cystometry procedure was performed in conscious rats 4 days after catheter implantation. A tube was connected to a pressure transducer (Powerlab, ADInstrument, Sydney, NSW, Australia) and an infusion pump (Promed-Tech., Bellingham, Massachusetts, USA) via a 3-way stopcock to record intravesical pressure (IVP) and to infuse saline into the bladder, respectively. Micturition volumes (MV) were recorded with a fluid collector connected to a force displacement transducer (Grass Inst. Co., Quincy, MA, USA). Room-temperature saline was infused into the bladder continuously at a rate of 0.2 ml/min. After the voiding pattern stabilized, the micturition cycles were recorded. The IVP and MV were recorded continuously with a data acquisition system (Chart software v5.5.6, ADInstrument) at a sampling rate of 2000 Hz.

**Fig. 1:**
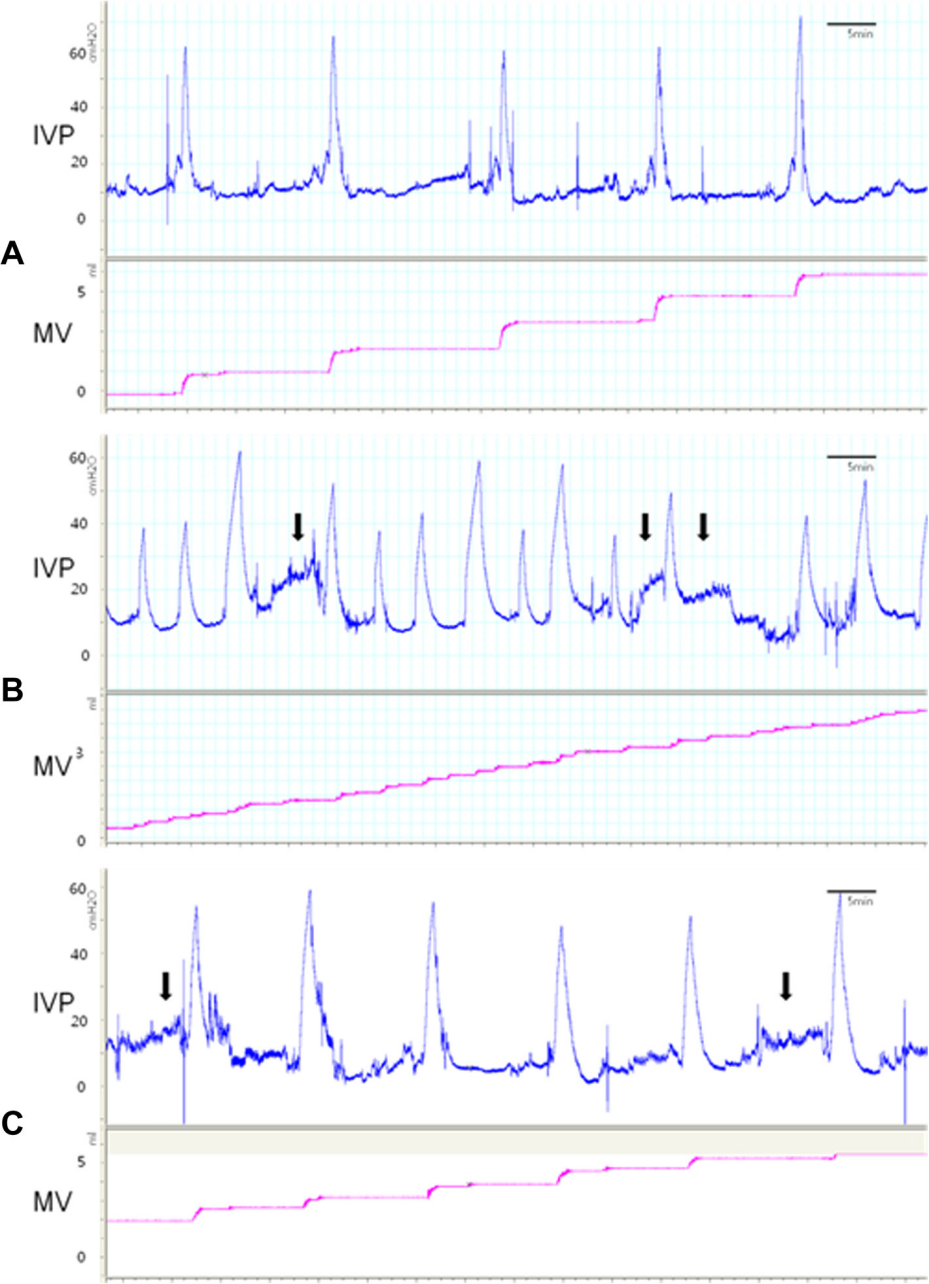
Representative cystometry results for the different study groups. Representative tracing of the cystometry parameters for intravesical pressure (IVP; cmH2O) and micturition volume (MV; ml). Black arrows represent non-voiding activity (NVA). **A**. Group I (controls). **B**. Group II (BOO rats). **C**. Group III (BOO rats administered 5-HMT)

In addition to MV, the following parameters were analyzed based on the cytometry results: micturition pressure (MP, maximum bladder pressure during micturition), basal pressure (BP, minimum bladder pressure between two micturitions), micturition interval (MI), micturition time (MT), and bladder weight (Fig. 1). Pressure values were compared by calculating the difference between BP values.

### Tissue preparation

After cystometry, the bladder of each rat was excised at the level of the ureteric orifices and proximal urethral and subsequently weighed. The urothelium and detrusor muscles were separated under microscopic vision with microscissors and microforceps. The separated layers from half of the bladder were stored in liquid nitrogen for reverse transcription polymerase chain reaction (RT-PCR) until needed. The other half of the bladder was used as soon as possible for primary cell cultures.

### RT-PCR of urothelium-expressed RNA

Total RNA was extracted from the frozen urothelium using Trizol (Invitrogen, Carlsbad, CA, USA). One milliliter of Trizol was added to the urothelium and homogenized in a 5-ml glass tube. The homogenate was transferred to a 1.0-ml tube and was mixed with 0.2 ml of 99 % chloroform (Sigma-Aldrich, St. Louis, MO, USA). After incubation for 5 minutes at room temperature, the homogenate was centrifuged at 13,200 × *g* for 10 minutes at room temperature. The supernatant was transferred to a clean tube, and 1 ml of isopropyl alcohol (Sigma-Aldrich) was added, followed by further centrifugation. After the supernatant was discarded, the pellet was mixed with 0.5 ml of diethylpyrocarbonate (DEPC; Sigma-Aldrich) and centrifuged at 13,200 × *g* for 10 minutes at room temperature. After discarding the supernatant, the pellet was dried at room temperature, dissolved with DEPC-treated water, and stored at −75 °C. cDNA was then prepared from 1 μg of random priming by using a First-Strand cDNA synthesis kit (Enzynomics, Korea), in accordance with the manufacturer’s protocol. It was incubated at 50 °C for 50 minutes, 70 °C for 10 minutes, and finally stored at −20 °C. The primer pair sequences are listed in Table 1. GAPDH was used as the reference gene. After an initial denaturation step at 95 °C for 5 min, an annealing procedure of 35 cycles (58 °C for 30 s), and an extension step (72 °C for 30 s and 72 °C for 5 min), were performed. The PCR products were separated on 1.2 % ethidium-containing agarose gels and photographed under an ultraviolet transilluminator. The band intensities were quantified by densitometry and qualified by Bio-ID (Vilber Lourmat, France).

**Table 1:** List of primer sequences used for RT-PCR

Gene	Primer sequence	Product
*GAPDH*	Forward: CAC GGC AAG TTC AAC GGC AC	189 bp
	Reverse: AGC GGA AGG GGC GGA GAT GA	
*BDNF*	Forward: CAT TCT TTC CCT CCC TCC TC	360 bp
	Reverse: CAG CTC CAC TTA GCC TCC AC	
*G-CSF*	Forward: TTG GCC ACT CTC TGG GTA TC	348 bp
	Reverse: GGT GAG CTG TCT CCA GGA AG	

### Urothelial cell culture

UCs were harvested from the bladders of each group using a previously described trypsin-based method (GIbco, Invitrogen, Carlsbad, CA, USA) [10]. The bladder was removed and placed in cold minimal essential medium (MEM; Invitrogen, Carlsbad, CA) supplemented with HEPES (2.5 g/l; Sigma, St. Louis, MO) and containing 1 % penicillin/streptomycin/fungi zone (PSF; Sigma). The bladder was turned inside out and incubated in dispase (2.5 mg/ml; Worthington Biochemical, Lakewood, NJ) overnight at 4 °C. UCs were gently scraped from the underlying tissue, placed in trypsin (0.25 % wt/vol; Sigma) for 10–15 min at 37 °C, and dissociated by trituration. Cells were suspended in MEM containing 10 % FBS (Invitrogen) and centrifuged at 416 × *g* for 10 min. After the supernatant was removed, the cells were suspended in keratinocyte media (bovine pituitary extract: 60 g/ml, hydrocortisone: 0.5 g/ml, insulin: 5 g/ml, epidermal growth factor: 0.1 ng/ml, gentamicin: 30 g/ml, amphotericin [Bio Whittaker, Walkersville, Maryland): 15 ng/ml, and human recombinant cholera toxin [Calbiochem, San Diego, California]: 8.3 ng/ml, 2 % FBS [Invitrogen]) with 1 % PSF, centrifuged again, and resuspended in fresh media. Cells were plated on collagen-coated glass coverslips at densities of 50–70 × 10^4^ cells/ml. Media was added after 4 hours of incubation at 37 °C following cell isolation and changed every other day. Cultured UCs were analyzed 48 hours after dissociation.

### RT-PCR of primary cultured UCs

RNA was prepared using the Cells-to-cDNA II kit (Ambion Europe Ltd, Huntingdon, UK). The lysates of the cell layer were processed according to the instructions until just before the RT step (i.e., 75 °C for 10 minutes, DNase I digestion at 37 °C for 15 minutes, and 75 °C for 5 minutes). The RNA preparations were stored at −20 °C for a short period. RT was also performed using the kit, according to the instructions using the random decamers provided as well as 5 μl of cell lysate (RNA). The resultant cDNA was diluted 5 times with water and stored at −20 °C. PCR was conducted using primer sets in accordance with the above-described method (Table 1).

### Data analysis

All statistical analysis was performed using SPSS, version 20.0 for windows (SPSS Inc., Chicago, IL, USA). A nonparametric Kruskal-Wallis test followed by a post-hoc test (Scheffe’s multiple comparison test) were performed for comparisons between the 3 groups. Differences were considered statistically significant when P < 0.05. The results are expressed as the mean ± sd.

## Results

### Animals

One hundred rats were used in the study: group I (20 rats), group II (40 rats), and group III (40 rats). Two rats each died in groups II and III after the BOO procedure. All control groups had a sham operation, and the cystometric investigations were performed after 4 weeks. Two rats in group II and 3 rats in group III were excluded because of failed BOO (i.e., no change in bladder weight and no difference in the voiding pattern compared to the control group). In the final analysis, groups I, II, and III had 20, 36, and 35 rats, respectively.

### Cystometry

Group II rats showed increased non-voiding activity [NVA; (number/10 voidings)] and bladder weight and decreased MV, MI, and MT relative to the control group (Table 2). Group III rats showed reduced NVA and bladder weight and increased MV and MI in comparison to group II rats.

**Table 2:** Comparison of cystometric parameters and bladder weight between the experimental groups. The results of various cystometric parameters and bladder weight for group I (control), group II (BOO rats), and group III (BOO rats after 5-HMT administration) are shown

Group (n)	TP-BP	MP-BP	MV	MI	MT	NVA	Bladder weight
I (20)	14.0 (a) ±0.97	55.4 (a) ±4.64	0.61 (a) ±0.06	64.7 (a) ±4.36	11.6 (a) ±1.33	0.15 (a) ±0.37	116.5 (a) ±8.26
II (26)	9.5 (b) ±6.95	55.2 (a) ±8.31	0.19 (b) ±0.05	27.4 (b) ±4.26	10.6 (b) ±0.85	2.36 (b) ±0.49	212.1 (b) ±12.00
III (25)	4.9 (c) ±3.51	49.24 (b) ±4.65	0.27 (c) ±0.64	39.3 (c) ±5.91	10.7 (b) ±1.45	1.31 (c) ±0.47	197.4 (c) ±10.66
p-value^1)^	0.000	0.001	0.000	0.000	0.017	0.000	0.000

TP, BP, MP: cmH_2_O; MV: mL; MT: seconds; bladder weight: mg; NVA (number/10 voidings)

The same letters indicate non-significant differences between groups based on Scheffe’s multiple comparison tests

1) Statistical significance among the different groups was tested by a nonparametric Kruskal-Wallis test

### RT-PCR results for the urothelium

**Fig. 2:**
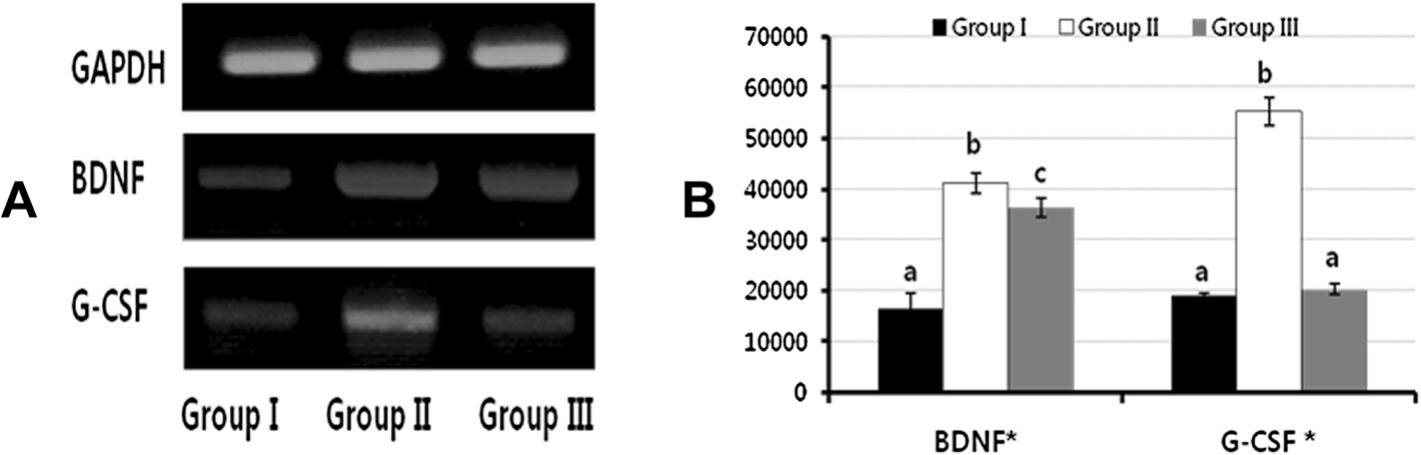
RT-PCR expression of BDNF and G-CSF in the urothelium. **A**. RT-PCR results. **B**. Densitometry measurements. *indicates statistical significance between groups (p < 0.001) as tested by a nonparametric Kruskal-Wallis test. The same letters indicate non-significant differences between groups based on Scheffe’s multiple comparison tests

Group II showed the highest expression of BDNF in the urothelium in comparison to groups I and III. G-CSF expression was significantly higher in group II relative to groups I and III, but did not significantly differ between groups I and III (Fig. 2). The injection of 5-HMT injection suppressed and partially suppressed the up-regulated mRNA expression of BDNF and G-CSF in BOO rats, respectively. Therefore, the changes in the voiding patterns were related to the expression of BDNF and G-CSF in the urothelium.

### Culturing and RT-PCR of UCs

**Fig. 3:**
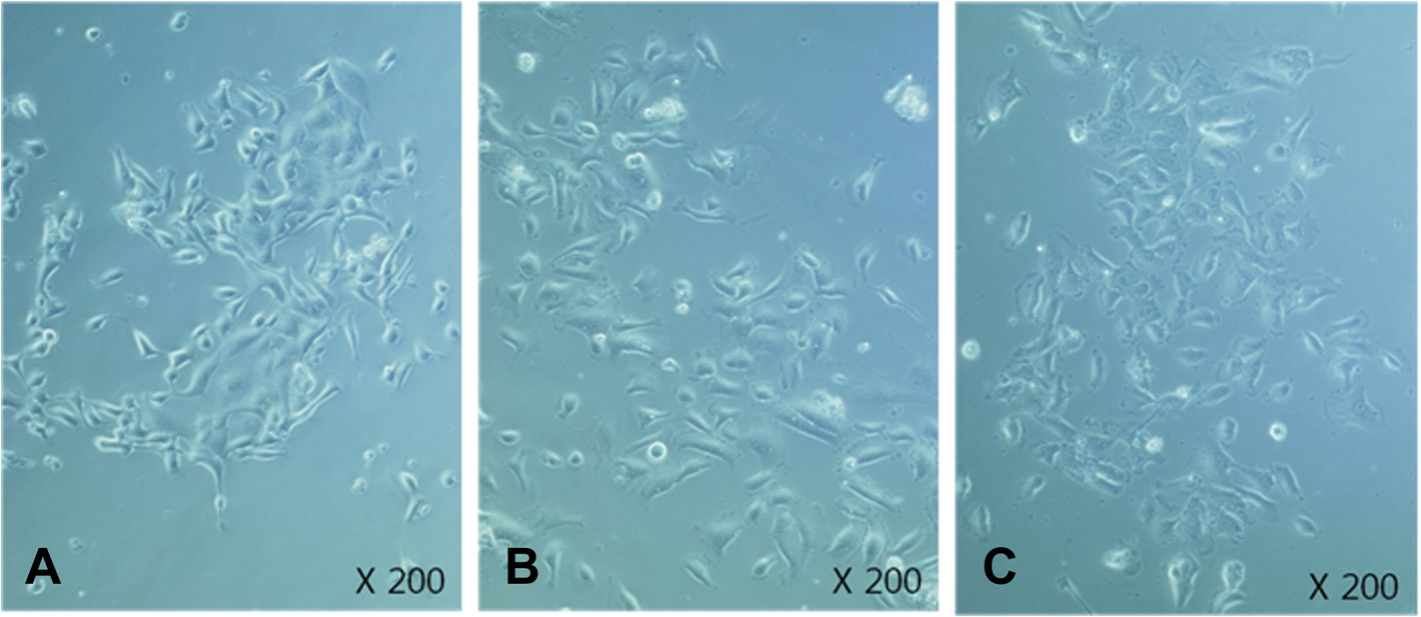
Cultured urothelial cells from each study group. **A**. Group I (controls). **B**. Group II (BOO rats). **C.** Group III (BOO rats administered 5-HMT)

**Fig. 4:**
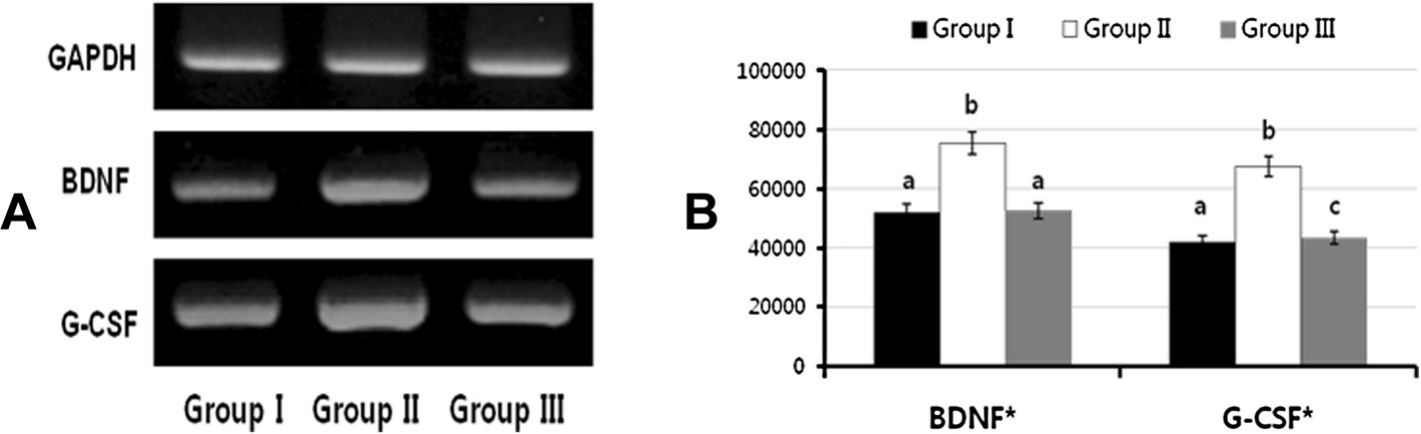
RT-PCR expression of BDNF and G-CSF in UCs. **A**. RT-PCR results. **B**. Densitometry measurements. *indicates statistical significance between groups (p < 0.001) as tested by a nonparametric Kruskal-Wallis test. The same letters indicate non-significant differences between groups based on Scheffe’s multiple comparison tests

In culture, the UCs proliferated and formed small islands (5–30 cells/island) (Fig. 3). No significant differences were observed in the number of living UCs between groups, indicating that no problems had resulted from the cell harvesting and culturing methods. The results for the cultured UCs were similar to those of the urothelium. The expression of BDNF and G-CSF was upregulated in the UCs of group II compared to that in the control, but was downregulated in group III (Fig. 4). The changes in both growth factors were related to the changes in the voiding patterns.

## Discussion

The changes in urothelial BDNF and G-CSF have not been previously observed in other OAB models. This might be due to the fact that neurogenic or myogenic BOO was assessed in these previous studies. This is an experimental model where, in particular, the BOO is “quickly” made instead of clinical OAB-BOO. Detrusor overactivity (DO) is the urodynamic hallmark of OAB, and the BOO rat can be useful for assessing potential pharmacotherapeutic concepts of DO in humans [11].

Moreover, various voiding patterns following BOO have been reported in this model. DO was identified and recorded as non-micturition-related increases in IVP [12], and a significant increase in MP was observed with this condition [2]. The common result of DO is a markedly shortened MI. The NVA in the present cystometry data consisted of small phasic contractions in the filling bladder. NVA is believed to represent the motor component of a motor/sensory system [13-15] and is accentuated in BOO. Moreover, NVA shows characteristics similar to DO in patients with OAB. Furthermore, tolterodine has been shown to significantly decrease the number and amplitude of NVA [16, 17]. In line with the cystometry results, the voiding pattern of groups II and III had characteristics similar to those for OAB and the treated condition. Hence, the present model is suitable as an experimental model.

In the storage phase, mechanical stretch stimulates the bladder afferents. This mechanosensory information is constantly sent to the central nervous system [18]. Alterations in afferent activity may lead to lower urinary tract dysfunction and is one of the possible mechanisms for OAB [18]. As the urothelium is a mechanosensory receptor, bladder function is influenced according to the changing state of the urothelium. Growth factors also may play a role in bladder function. Moreover, growth factors have been found to play a crucial role in various cellular processes such as proliferation, differentiation, migration, apoptosis, and neurodegeneration [19]. Previous research has examined the correlation between OAB and NGF. It was observed that increased NGF expression in the bladder may contribute to storage symptoms (e.g., urgency and frequency) in patients with OAB [20, 21]. In addition, NGF expression in the bladder was found to be associated with the clinical symptoms of OAB [22]. It has become clear that NGF modulates neuronal function along micturition pathways, is involved in multiple bladder pathologies, and serves as a urinary marker of OAB [21]. BDNF, a member of the NGF family, is primarily present in the sensory neuronal cell body in the dorsal root ganglion and is involved in sensory neuronal activation in a variety of animal models [23]. Of note, BDNF synthesis has been shown to be stronger in the bladder after chronic bladder inflammation [5]. Moreover, in patients with bladder pain syndrome/interstitial cystitis, the urinary concentration of BDNF was high at baseline but was significantly reduced after botulinum toxin administration to the bladder trigone [24]. Consistent with this previous finding, BDNF sequestration improved voiding function in rats with chronic cystitis [5]. Accordingly, BDNF has been found to be a sensitive indicator of the resolution of lower urinary tract disorders by therapeutic management [4].

On the other hand, the G-CSF receptor is markedly upregulated in neurons during cerebral ischemia and has direct effects on neurons, including the reduction of neuronal apoptosis and the stimulation of endogenous neural progenitors [25]. A further mechanism of action for G-CSF in stroke is the mobilization of bone marrow-derived stem cells to participate in neurogenesis and angiogenesis [26]. G-CSF has also been shown to have neuroprotective effects after peripheral axotomy [27]. Previous studies have also investigated G-CSF in terms of the diagnosis and treatment of bladder cancer. However, no prior research has confirmed the relevance of G-CSF to voiding function. In the present study, the functions of G-CSF and BDNF in the urothelium were evaluated at the tissue and cellular level by specifically using only the urothelium and UCs. A similar upregulation of G-CSF and BDNF expression and subsequent downregulation following 5-HMT treatment was observed in BOO rats for both the urothelium and UCs. BOO has been found to induce inflammation, multiple nerve injury, and changes in nerve density [28]. One effect stemming from neural damage inflicted by BOO might be an increase in the neuroprotective activity of G-CSF. Nonetheless, a clear mechanism, in terms of changes in the expression of these growth factors and their attendant roles, remains unknown.

DO might be attributable to an increase in afferent activation. The proximity of the afferent nerves to the urothelium suggests that chemicals released by the urothelium might alter afferent excitability. Hence, the urothelium could be important in the manifestation of voiding symptoms, and changes in the expression of various urothelial receptors might play a key pathophysiological role in OAB occurrence. At therapeutic doses, muscarinic antagonists do not seem to inhibit bladder contractility [29]. Hence, OAB appears to be affected by factors other than those involved in bladder muscle contractile dysfunction, thus also suggesting that the urothelium potentially plays an important role in voiding function. Based on these findings and additional previous ones, the investigation of urothelium-expressed G-CSF and BDNF in voiding function seemed suitable for further exploring urothelial function. In particular, previous studies have indicated that NGF, BDNF, prostaglandins, cytokines, and C-reactive protein may be suitable biomarkers of OAB [4, 30-32]. Furthermore, the evaluation of urine samples from OAB patients revealed that G-CSF is upregulated by several different molecules [33]. Within the present experimental context, the use of the urothelium and cultured UCs from the bladder allowed a more robust examination of the changes in these growth factors. In both rats and humans, the M1 to M5 muscarinic receptors are distributed throughout the urothelium [34], and Kullmann et al. detected the expression of all 5 muscarinic receptors subtypes in UCs [35]. We chose 5-HMT as a non-selective agent that could serve as a competitive blocking agent for all muscarinic receptors [6, 36]. Following 5-HMT administration, both growth factors were altered in the control as voiding dysfunction improved. Because the changes in the growth factors were observed in the urothelium and UCs, they will also presumably affect bladder function. Hence, based on the present findings, the urothelium may not only function simply as a mechanosensory receptor, but also play an important role in bladder function. Our study also provides new evidence that 5-HMT has a significant effect on the expression of growth factors in addition to its antimuscarinic tendencies.

A limitation of the present study is that quantitative tests could not be performed because of the small number of UCs. However, the use of RT-PCR with the cell culture allowed an examination of the changes in the characteristics of the UCs. The results showed that BDNF and G-CSF expression was altered in each group and that these alterations had a significant relationship with voiding function.

## Conclusions

BDNF and G-CSF expressions were increased in BOO rats and decreased following 5-HMT administration in both the urothelium and UCs. The expression of BDNF and G-CSF in the urothelium might be related to voiding dysfunction in OAB patients. As a therapeutic agent for OAB, 5-HMT improves voiding dysfunction. Based on the present findings, the urothelium plays an important role in the manifestation of voiding symptoms.

## Abbreviations

5-HMT: 5-Hydroxymethyl tolterodine
BP: Basal pressure
BOO: Bladder outlet obstruction
BDNF: Brain derived-neurotrophic factor
UCs: Cultured urothelial cells
G-CSF: Granulocyte-colony stimulating factor
IVP: Intravesical pressure
MEM: Minimal essential medium
MI: Micturition interval
MP: Micturition pressure
MT: Micturition time
MV: Micturition volume
NGF: Nerve growth factor
NVA: Non-voiding activity
OAB: Overactive bladder
RT-PCR: Reverse transcription polymerase chain reaction
